# Gyroscope-constrained magnetometer PDR/Wi-Fi indoor positioning algorithm

**DOI:** 10.1371/journal.pone.0335277

**Published:** 2025-10-24

**Authors:** Ruiyi Tang, Chengkai Tian

**Affiliations:** 1 Xi’an Jiaotong University, Xi’an, Shaanxi, P.R.China; 2 Xi’an University of Posts & Telecommunications, Xi’an, Shaanxi, P.R.China; University of Lahore - Raiwind Road Campus: The University of Lahore, PAKISTAN

## Abstract

To address the issue of low precision in sensor data measured by smartphones, we propose a gyroscope-constrained magnetometer Pedestrian Dead Reckoning (PDR)/Wi-Fi indoor positioning algorithm, focusing on improving the PDR heading angle. We utilize the heading angle constrained by the gyroscope and magnetometer and enhance fingerprint data using Kriging interpolation, effectively doubling the signal fingerprint density. We combine the optimized PDR algorithm and Wi-Fi fingerprint positioning results through an Extended Kalman Filter. Experimental results show that the traditional PDR algorithm has an average positioning error of 2.02 meters, with 90% of errors below 3.71 meters. The improved PDR algorithm reduces the average positioning error to 1.07 meters, with 90% of errors below 2.12 meters. Integrating Wi-Fi and the improved PDR algorithm further reduces the average positioning error to 0.71 meters, with 90% of errors below 1.42 meters.

## 1. Introduction

In indoor positioning, each standalone positioning technology offers unique advantages and limitations. Given the complexity of different scenarios and environments, relying on a single method for smartphone positioning presents numerous challenges. Multi-source fusion positioning technology has emerged as an effective solution to fully exploit the strengths of various positioning systems and enhance indoor positioning performance [1,2]. This technology integrates complementary sensor data from multiple sources, significantly improving positioning accuracy [3,4]. By leveraging the advantages of different sensors, it compensates for the deficiencies of individual systems, providing more stable and precise results for indoor positioning. Multi-source fusion technology effectively addresses the complexities and variability of different environments, achieving more reliable and efficient smartphone positioning. Multi-source fusion has become a core direction in the development of indoor navigation technology. In smartphone indoor positioning, fusion methods often encompass a combination of Wi-Fi, Bluetooth [5], geomagnetic [6], and Pedestrian Dead Reckoning (PDR) [7,8]. The integration of these technologies dramatically enhances positioning accuracy and stability. Each technology provides location information through different mechanisms with unique strengths and limitations. Common strategies include Kalman filtering and particle filtering to fully leverage their advantages and mitigate their shortcomings [9,10]. These methods effectively integrate data from various technologies, thereby improving the accuracy and stability of indoor positioning.

In the research on Wi-Fi and PDR fusion positioning, Chen et al. [7] employed Kalman filtering to integrate Wi-Fi fingerprint positioning results with PDR positioning results. This approach reduced the PDR positioning error and enhanced the stability of Wi-Fi positioning. Utilizing the prediction and update mechanisms of Unscented Kalman Filtering more effectively managed the uncertainty and noise in sensor data, significantly improving the accuracy and stability of the fusion positioning. Wang Zhaoyuan et al. [8] integrated Wi-Fi, geomagnetic, and PDR technologies comprehensively. To address the particle degeneration problem, they introduced a sequential particle weighting method. Additionally, they reduced computational complexity by updating particle weights through region partitioning. This fusion positioning method achieved accuracy within one meter in an indoor corridor environment. This method successfully combined the unique advantages of different sensors, significantly improving positioning accuracy and stability. Moreover, the optimized particle filtering algorithm effectively reduced computational load, enhancing both the practicality and stability of the algorithm. Liu Y [6] proposed a three-dimensional floor positioning method based on the fusion of PDR and geomagnetic information. By analyzing acceleration signals together with barometric pressure variations, this method provides a theoretical basis for identifying whether a pedestrian is ascending or descending stairs, thereby supporting the construction of a geomagnetic reference map [11]. Furthermore, the particle filtering technique was used to finely correct the PDR results, ensuring the precision of the pedestrian’s movement trajectory. By combining the advantages of both PDR and geomagnetic information, this method improved the accuracy and stability of floor positioning.

## 2. Related work

### 2.1 Wi-Fi indoor positioning algorithm

**Fig 1:**
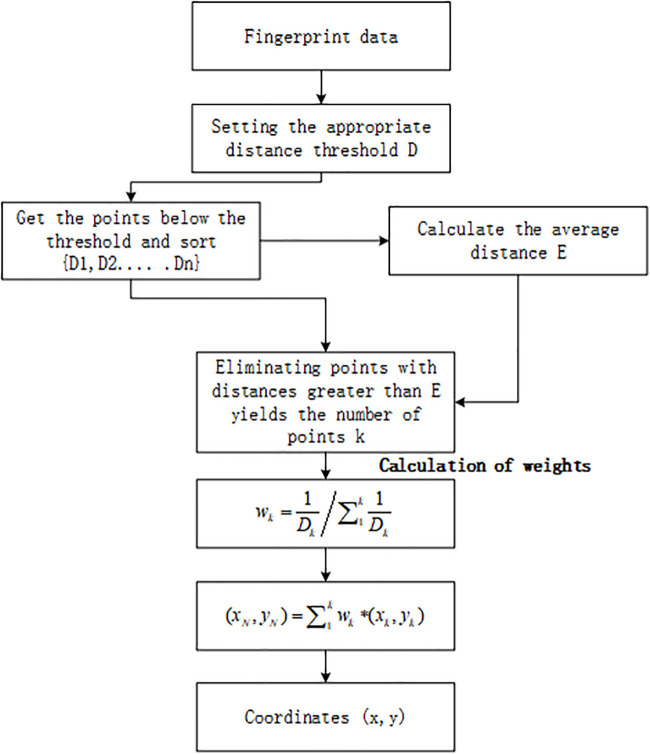
EWKNN algorithm flow chart. This algorithm can obtain an adaptive dynamic position.

Commonly used positioning algorithms are deterministic matching algorithms and probabilistic matching algorithms. For example, NN(Nearest Neighborhood), KNN (K Nearest Neighborhood), WKNN(Weighted K Nearest Neighborhood), and VWKNN(Variation Weighted K Nearest Neighborhood) are typical deterministic matching algorithms. The naive Bayes algorithm and the improved algorithm are probability-matching algorithms. The KNN algorithm uses the mean as the positioning result to ignore the distance between the undetermined point and the different adjacent points [12,13]. The WKNN algorithm weakens the adverse effect of the estrangement point on the final positioning, cannot dynamically determine the value of K, and cannot find the optimal K value [14]. VWKNN [15]considers the degree of signal dispersion at the reference point and calculates the dispersion of each reference point as a weight. The above methods need to consider the influence of the K value on the positioning results. The fixed K value will reduce the mobility and availability of the algorithm. Therefore, this paper chooses to use the EWKNN algorithm for Wi-Fi positioning to provide initial bit value and correction for the PDR algorithm. The EWKNN algorithm flow is shown in Fig 1.

Fig 1 is the algorithm flow of EWKNN. Based on WKNN, the algorithm selects the number of reference points whose Euclidean distance is less than the threshold distance as n and sorts it to calculate the average distance. D in the image is the distance threshold, and {D1, D2, D3…Dn} are the points with Euclidean distances below the threshold sorted accordingly. By eliminating the points that are more significant than the mean distance, *k* points whose distance is less than the mean value are obtained. The *k* value is dynamically adjusted according to the mean value of the distance between each point.

After calculating the weight $w_{k}$ of each point based on distance weighting, substitute the weight and coordinates $\left(x_{k},y_{k}\right)$ of each point into the formula in the graph $(x_{N},y_{N})=\sum {\mathrm{\backslash nolimits}}_{1}^{k}w_{k}*(x_{k},y_{k})$ to calculate the current position coordinates (x, y).

### 2.2 PDR indoor positioning algorithm

PDR is a relative positioning technology using smartphones’ built-in MEMS inertial measurement unit [16], which can continuously track the two-dimensional position of pedestrians. As an autonomous localization method that does not rely on external signals, PDR calculates and updates the pedestrian’s position at the next moment by combining the position information of the previous moment (X_t_, Y_t_) with the heading and displacement data provided by the inertial sensor (X_t + 1_, Y_t + 1_) [17]. This positioning method mainly depends on the sensor data inside the mobile phone, which realizes the continuous tracking of pedestrian position. The specific process is calculated as follows:


$$\{\begin{array}{c} {}*20cX_{t+1}=X_{t}+step\_len_{t}*\cos\alpha_{t}\\ Y_{t+1}=Y_{t}+step\_len_{t}*\sin\alpha_{t} \end{array}$$ (1)

There, step _ len denotes step length and heading angle. PDR’s core problem lies in step length, step frequency, and heading angle [18,19]. This section will introduce the algorithm principle of three aspects. First, the step frequency is detected by zero-crossing detection and trough pairing principle to improve the accuracy of step frequency detection. A step length calculation method is proposed to consider acceleration and peak valleys in the gait cycle. In addition, the heading is updated using geomagnetic calculation and gyroscope correction.

(1) Step frequency detection

In a complete gait cycle, the acceleration will appear in apparent peaks and troughs, and the two usually appear in pairs. In addition, between the trough and the peak, the acceleration will go through a zero.

**Fig 2:**
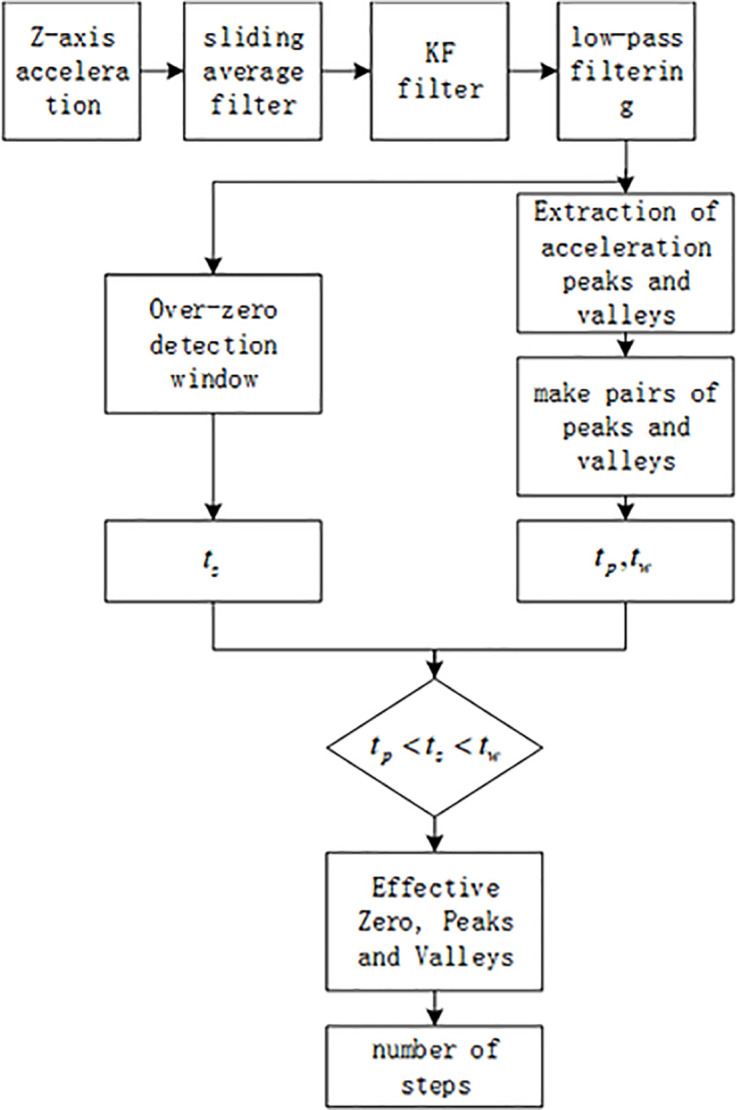
Step frequency detection algorithm. Whether a step is taken is determined by the relationship between the zero-crossing time, peak time points, and trough time points.

Based on this feature, this paper takes the acceleration passing through the trough peak in pairs and the zero crossing as the basis or principle for judging pedestrians to take a step. The algorithm flow is shown in Fig 2. After multiple continuous filtering of the acceleration signal, the above peak-valley and zero-crossing detection algorithms are used for the solution.

When zero timestamp $t_{z}$, peak timestamp $t_{p}$, and trough timestamp $t_{w}$ have the relationship $t_{p}<t_{z}<t_{w}$, it is considered a valid trough and peak, completing a step.

(2) Step length calculation

Step length is affected by many factors, such as height, age, gender, and pace [20]. However, in practical applications, it is often impossible to directly obtain the personal information of pedestrians [21]. In order to solve this problem, we can use the characteristics of the sensor data itself to construct the step size model. This way, relatively accurate step length information can be estimated from sensor data, even without additional personal information. The step size will change significantly when the pedestrian’s walking state changes, such as from fast to slow walking. This change will also be reflected in the acceleration characteristics because there is a specific correlation between acceleration and step length. In order to estimate the step size more accurately, we can use the relationship between the acceleration characteristics to model and analyze. This way, even if the pedestrian’s walking state constantly changes, it can accurately understand its movement state. The Weinberg [22] model holds that there is a positive correlation between the pedestrian’s step length and movement amplitude. Meanwhile, the body’s movement amplitude is related to the variation range of body acceleration, which is calculated by the difference between the peak and trough values. The formula is as follows:


$$L=K^{4}\sqrt{a_{\max}-a_{\min}}$$ (2)

*a*_max_ is the maximum value of acceleration within a gait cycle, and *a*_min_ is the minimum value of acceleration within a gait cycle. K is the model parameter, and L is the step length.

The Kim [23] model is an estimation model that takes into account all acceleration information within a single step of a pedestrian. Compared with the Weinberg model, it reduces a certain amount of acceleration noise interference. The formula is as follows:


$$L=K^{3}\sqrt{\frac{\sum {\mathrm{\backslash nolimits}}_{i=1}^{N}\left|a_{i}\right|}{N}}$$ (3)

Among them, *a*_i_ represents the acceleration at each sampling point within a gait cycle, N denotes the number of sampling points, K is the model parameter, and L stands for the step length.

Article [18] makes improvements based on Weinberg’s (model/theory) and optimizes the error problem caused by fixed coefficients. The formula is as follows:


$$L=(m*\frac{\text{1}}{t})K^{4}\sqrt{a_{\max}-a_{\min}}$$ (4)

Among them, L is the improved estimated step length, *a*_max_ and *a*_min_ are the maximum and minimum values of the resultant acceleration in each step, and m and k are coefficients.

In this paper, an adaptive step size estimation model is proposed. This model combines multiple factors, including stride frequency, the ratio of the sum of accelerations in the gait cycle to stride frequency, and the difference between the peak and trough of acceleration.

This adaptive feature makes the step size estimation more accurate and improves the robustness of the model in the face of different walking modes. The formula is as follows:


Len=a*sf+b*∑\nolimits1j|Acct|/|Acct|f\nulldelimiterspacef+c*Acc_diff+d (5)

Among them, *Len* is the step length, *sf* is the step frequency, ∑\nolimits1j|Acct|/|Acct|f\nulldelimiterspacef is the sum of the acceleration ratio frequency in a gait cycle, and *Acc _ diff* is the difference between the acceleration peaks and valleys in a gait cycle. The coefficients of each characteristic parameter in the equation of *a, b, c, d.* The coefficients were obtained via multiple linear regression.

(3) Heading angle estimation

**Fig 3:**
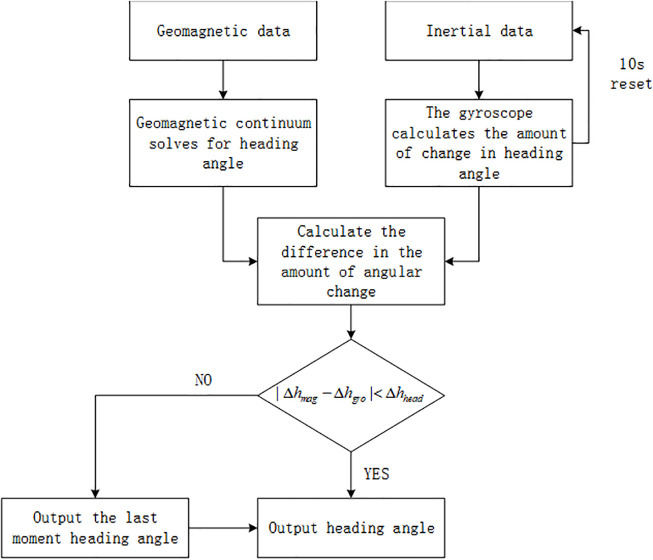
Heading angle estimation algorithm. A real-time heading angle is provided by the magnetometer, and the accurate heading angle is obtained by constraining the variation of the magnetometer with the gyroscope.

This paper proposes a heading estimation algorithm for gyroscope correction geomagnetic. It is proposed that the heading angle of magnetic calculation be corrected by using the variation of the gyroscope in a short time. The algorithm flow is shown in Fig 3.

This method uses a gyroscope to correct the heading of the magnetometer, comparing the differential calculation between the change in the magnetometer ($\Delta h_{mag}$) and the change in the gyroscope ($\Delta h_{gro}$) with the change in the actual heading angle ($\Delta h_{head}$), where $\Delta h_{head}$ is the difference in the actual heading angle between the last two moments. When the difference between the magnetometer and the gyroscope is less than the actual change in heading angle, it is considered that the magnetometer is not disturbed and directly outputs the result as the actual heading angle. When it is greater than the actual change in heading angle, it is considered that the magnetometer is abnormal, the calculated heading is inaccurate, and the magnetometer’s heading angle from the previous moment is output as the actual heading angle.

**Fig 4:**
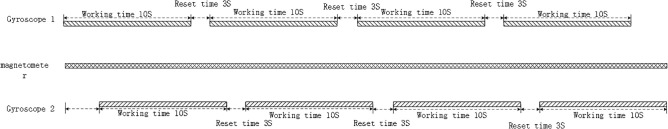
Gyroscope magnetometer working sequence diagram.

In pedestrian walking, the gyroscope is reset once every ten seconds to provide more accurate gyroscope data for the heading angle calculated by the constraint magnetometer. The gyroscope only needs to detect the change in the pedestrian heading angle and does not need to carry out complex coordinate transformation. Since a single gyroscope has an empty window period of not less than 4s after reset and before stable output, the gyroscope will not be able to constrain the heading angle of the magnetic force during the geomagnetic mutation and pedestrian heading angle change during the empty window period. It is improved to the heading angle calculation of the magnetometer constrained by double gyroscopes to ensure that there is a gyroscope that can be used to constrain the heading angle of the magnetic solution at any time. The working sequence of the magnetometer and gyroscope is shown in Fig 4.

As shown in Fig 4, the operation of gyroscope 2 is delayed by 4s so that at least one gyroscope can constrain the operation of the magnetometer for all working hours. Each gyroscope adopts the constraint algorithm shown in Fig 3. When only gyroscope 1 works, gyroscope 1 is used for constraint. When two gyroscopes are working, gyroscope 1 is still used. When only gyroscope 2 is used, gyroscope 2 is used for constraint.

(4) The position model proposed in this paper

**Fig 5:**
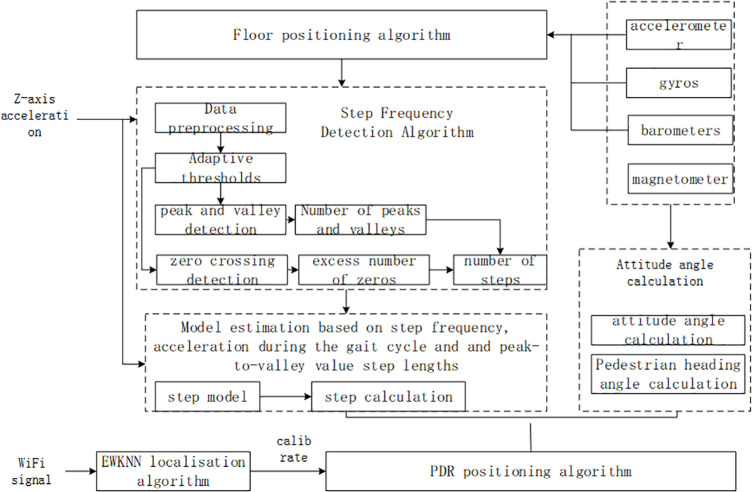
WiFi/ PDR fusion localization algorithm.

This paper proposes an indoor positioning technology based on Wi-Fi/ PDR fusion. In the case of plane walking, the positioning results of Wi-Fi signals [24] are used to fuse the improved PDR (Pedestrian Dead Reckoning)algorithm to achieve accurate position estimation. As shown in Fig 5:

By using EKF(Extended Kalman Filter), the nonlinear PDR positioning model is expanded by the Taylor series [25,26], and the terms above the second level are omitted to obtain an approximate linearized model to obtain the fusion positioning of PDR and WiFi positioning results. The dynamic position information model is established as follows:

① State transition equation:

$$X_{n}=\left[\begin{array}{c} {}*20cx_{n}\\ y_{n}\\ \theta_{n} \end{array}\right]=\left[\begin{array}{c} {}*20cx_{n-1}+l_{n-1}\sin\theta_{n-1}\\ y_{n-1}+l_{n-1}\cos\theta_{n-1}\\ \theta_{n-1}+\Delta \theta \end{array}\right]+r_{1}$$ (6)

Where $r_{1}$ is the process noise of non-Gaussian distribution of the state transition equation; $\left(x_{n},y_{n}\right)$ is the fusion positioning result of the nth step, $\left(x_{n-1},y_{n-1}\right)$ is the fusion positioning result of the n-1th step, $\theta_{n}$ is the heading angle of the nth step, $\theta_{n-1}$ is the heading angle of the n-1th step, and Δθ is the estimated increment of the direction angle.

② Observation equation:

$$Y_{n}=[x_{n},y_{n},l_{n},\theta_{n}{]}^{T}+r_{2}$$ (7)

Among them, r2 is the observation noise, $\left(x_{n},y_{n}\right)$ is the Wi-Fi fingerprint positioning result, and $l_{n}$ is the observation step size.

③ Initial state:

The initial position is the positioning result $\left(x_{0},y_{0}\right)$ obtained by the EWKNN positioning model, and the initial covariance is P_1_. Then the priori estimate of the system is:


$$X_{n}^{\prime }=F_{n}X_{n-1}$$ (8)

$$P_{n}^{\prime }=F_{n}P_{n-1}F_{n}^{T}+R_{1}$$ (9)

where, $F_{n}$ is the state transition matrix, and R1 is the covariance matrix of process noise r1.

④ Update phase:

The updated Kalman gain of step n is as follows:


$$K_{n}=P_{n}^{\prime }H^{T}(HP_{n-1}H^{T}+R_{2}{)}^{-1}$$ (10)

where H is the observation matrix and R2 is the covariance matrix of process noise r2.

⑤ Output results:

The state and covariance matrix are updated by the observation variable Yn, and the posteriori estimation of the system is shown as follows:


$$X_{n}=X_{n}^{\prime }+K_{n}(Y_{n}-Y_{n}^{\prime })$$ (11)

$$P_{n}=(1-K_{n}H)P_{n}^{\prime }$$ (12)

The output *X_n_* is updated circularly to obtain the fusion positioning coordinate position $\left(x_{n},y_{n}\right)$.

## 3. Experimental setup

(1) Establish a Wi-Fi signal database

**Fig 6:**
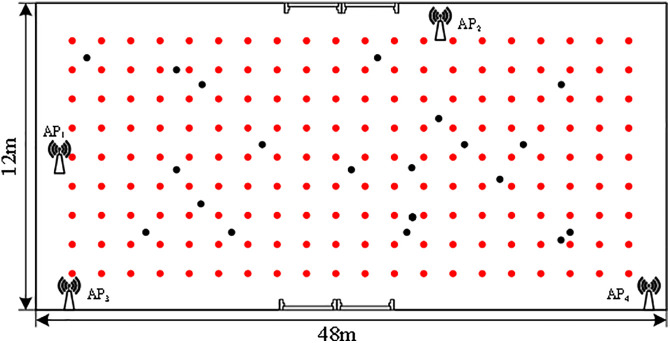
Data acquisition schematic diagram.


Four APs are deployed in the lobby of the first floor of the experimental building, and the acquisition grid is divided into 0.6 * 0.6m grids as the acquisition points. The data acquisition method of each AP at the reference point is shown in Fig 6. Each red point is a reference point, and the black is the random test point. Each reference point continuously collects one minute of data at a frequency of 1 Hz. After Gaussian filtering, the average value is recorded as the fingerprint of the point, and the location fingerprint(LF) database structure is Equation (1)

$$LF=\left[\begin{array}{cccccc} {}*20cx_{1} & y_{1} & rssi_{1}^{1} & rssi_{1}^{2} & \cdots & rssi_{1}^{n}\\ x_{2} & y_{2} & rssi_{2}^{1} & rssi_{2}^{2} & \cdots & rssi_{2}^{n}\\ \vdots & \vdots & \vdots & \vdots & \ddots & \vdots \\ x_{m} & y_{m} & rssi_{m}^{1} & rssi_{m}^{2} & \cdots & rssi_{m}^{n} \end{array}\right]$$ (13)

In the formula, LF is an offline fingerprint database, which contains the coordinates of each reference point and the RSS value of each AP at that point, and (xm,ym) is the physical location of the *m*th point. $rssi_{m}^{n}$ is the signal strength of the nth AP received at the *m*th reference point.

(2) WiFi Fingerprint Data Augmentation

The Wi-Fi signal acquisition mentioned in the previous section is a fingerprint database collected at an interval of 0.6m in the indoor environment. At present, there are two main ways to improve the accuracy of positioning. One is to optimize the fingerprint algorithm, and the other is to optimize the fingerprint data, filter the fingerprint data of each AP, or interpolate the data to improve the data density. This study will interpolate the original data to improve the fingerprint density. The commonly used interpolation algorithms include nearest neighbor interpolation, inverse distance weighted interpolation, and Kriging interpolation. The Wi-Fi fingerprint database is arranged in physical coordinates, and the Wi-Fi fingerprint database diagram is established by different interpolation methods. By calculating the index parameters of the above three interpolation algorithms, a reasonable choice is made.

The appropriate algorithm is selected by quantitative analysis of the characteristics of the three. In order to obtain the difference between the data sets before and after interpolation processing, the two data sets are compared. These two data sets are the original data set (including the Wi-Fi signal strength of the original measurement) and the interpolated data set. The Wi-Fi signal strength values of each corresponding position in the two data sets are subtracted, and the difference is calculated. The difference is the residual deviation value based on the received signal strength. In addition, in order to quantify this degree of deviation, the standard deviation of these deviation values is calculated, and the maximum deviation and the minimum deviation are compared in dBm. The results are shown in Table 1.

**Table 1:** Performance of interpolation algorithm.

Interpolation method	minimum deviation	maximum deviation	standard deviation
Nearest neighbor interpolation	−7.83	8.93	0.89
Inverse distance weighting	−9.66	7.65	2.66
Kriging	−11.3	10.26	1.61

From the data in Table 1, it can be observed that the Kriging interpolation method demonstrates more pronounced data deviation compared to the Inverse Distance Weighting interpolation method and the Nearest Neighbor interpolation method. This suggests that the raster data generated by Kriging interpolation may be slightly less effective in terms of the concentration and dispersion of values. However, in contrast to Inverse Distance Weighting and Nearest Neighbor methods, it exhibits more intuitive and easily identifiable characteristics, making the data more conducive to feature matching.

(3) Real-Time Inertial Data Acquisition and Test Path

**Fig 7:**
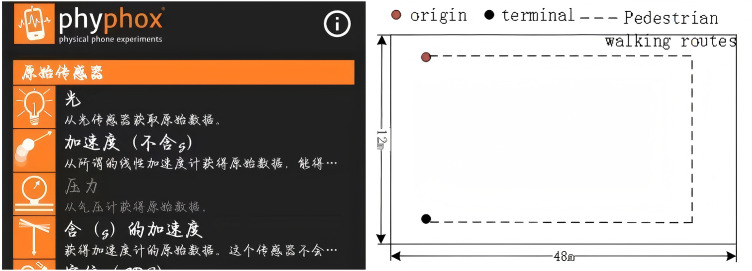
Phyphox interface and collection route. (a) phyphox user interface. (b) Pedestrian actual data collection route.

The experimental scene is the first-floor hall of the experimental teaching building of the school. The indoor environment is shown in Fig 7, using the accelerometer, magnetometer, and gyroscope integrated with the Glory V30 mobile phone. Using phyphox data acquisition software, data output for CSV and EXCEL two data formats. The software interface is shown in Fig 7(a):

In the data acquisition stage, the software collects pedestrian motion state information, and the frequency is set to 50 Hz. The pedestrian movement trajectory is shown in the Fig 7(b) The pedestrian’s handheld mobile phone walks along the established route to collect data and saves the data in EXCEL format for the simulation platform. The hardware configuration of the simulation platform is AMD Ryzen7 5800H CPU 16G memory, NVIDIA GeForce RTX3060Ti 8GB GPU, and the software configuration is 64-bit Windows 10 professional version and Matlab 2019.

## 4. Results

### 4.1 Wi-Fi fingerprint positioning results

In Wi-Fi fingerprint positioning, the EWKNN algorithm is used to locate the fingerprint. In order to verify the algorithm, 160 points are randomly collected in the environment of collecting fingerprint database by the same method, and the four indexes of maximum error, minimum error, average error, and cumulative distribution function (CDF) of 160 points are calculated. The advantages and disadvantages of KNN, WKNN, and EWKNN are compared. In another set of experiments, the positioning performance of KNN, WKNN, and EWKNN algorithms after interpolation is compared using the same test points.

It can be seen from Table 2 that KNN, WKNN, and EWKNN positioning errors, KNN positioning error is the largest, WKNN positioning error is more minor than KNN, EWKNN positioning error is the smallest, and KNN and WKNN results are optimal when K = 4.

**Table 2:** Error of different positioning algorithms under raw data.

localization algorithm	Average positioning error/ m	Maximum error/ m	Minimum error/ m
KNN algorithm	2.29	3.97	1.33
WKNN algorithm	2.05	3.52	0.89
EWKNN algorithm	1.75	2.89	0.59

**Fig 8:**
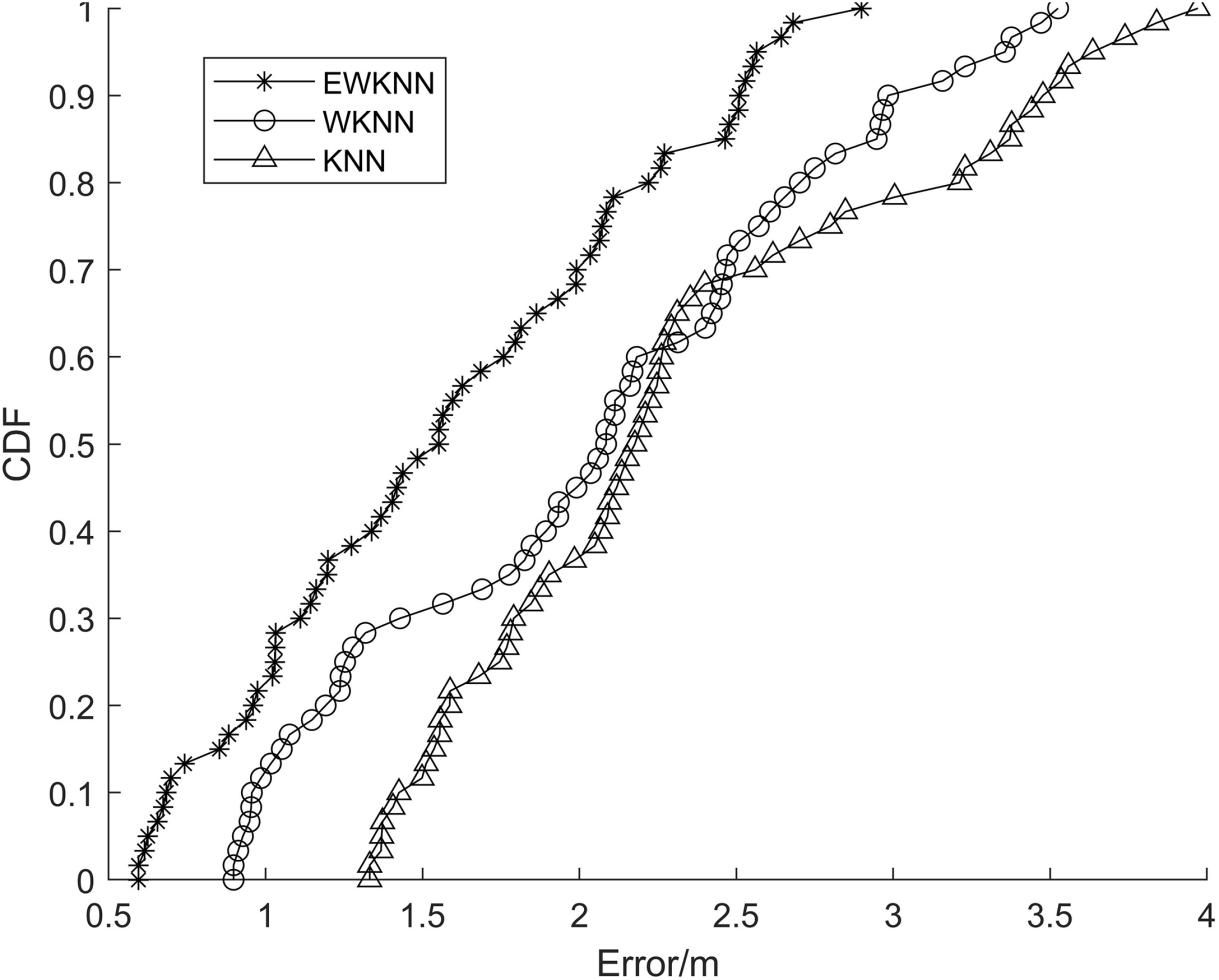
The cumulative distribution function of the original fingerprint error.

The error probability statistical analysis of the above three algorithms is carried out, and the cumulative distribution of positioning error is shown in Fig 8. It can be seen from the figure that the probability that the positioning accuracy of the EWKNN algorithm is better than 2.5m is 88%, and the probability that the accuracy of WKNN and KNN algorithms is better than 2.5m is 76% and 69%, respectively.

In order to better verify the conclusion in 2.1, the EWKNN algorithm is tested on the fingerprint database enhanced by three different interpolation algorithms. Table 3 shows the experimental results.

**Table 3:** The positioning error of EWKNN in different fingerprint databases.

Interpolation algorithm	Average positioning error/ m	Maximum error/ m	Minimum error/ m
Kriging interpolation	1.26	1.95	0.44
Inverse distance interpolation	1.52	2.36	0.53
Nearest neighbor interpolation	1.72	2.52	0.72
The original fingerprint database	1.75	2.89	0.59

**Fig 9:**
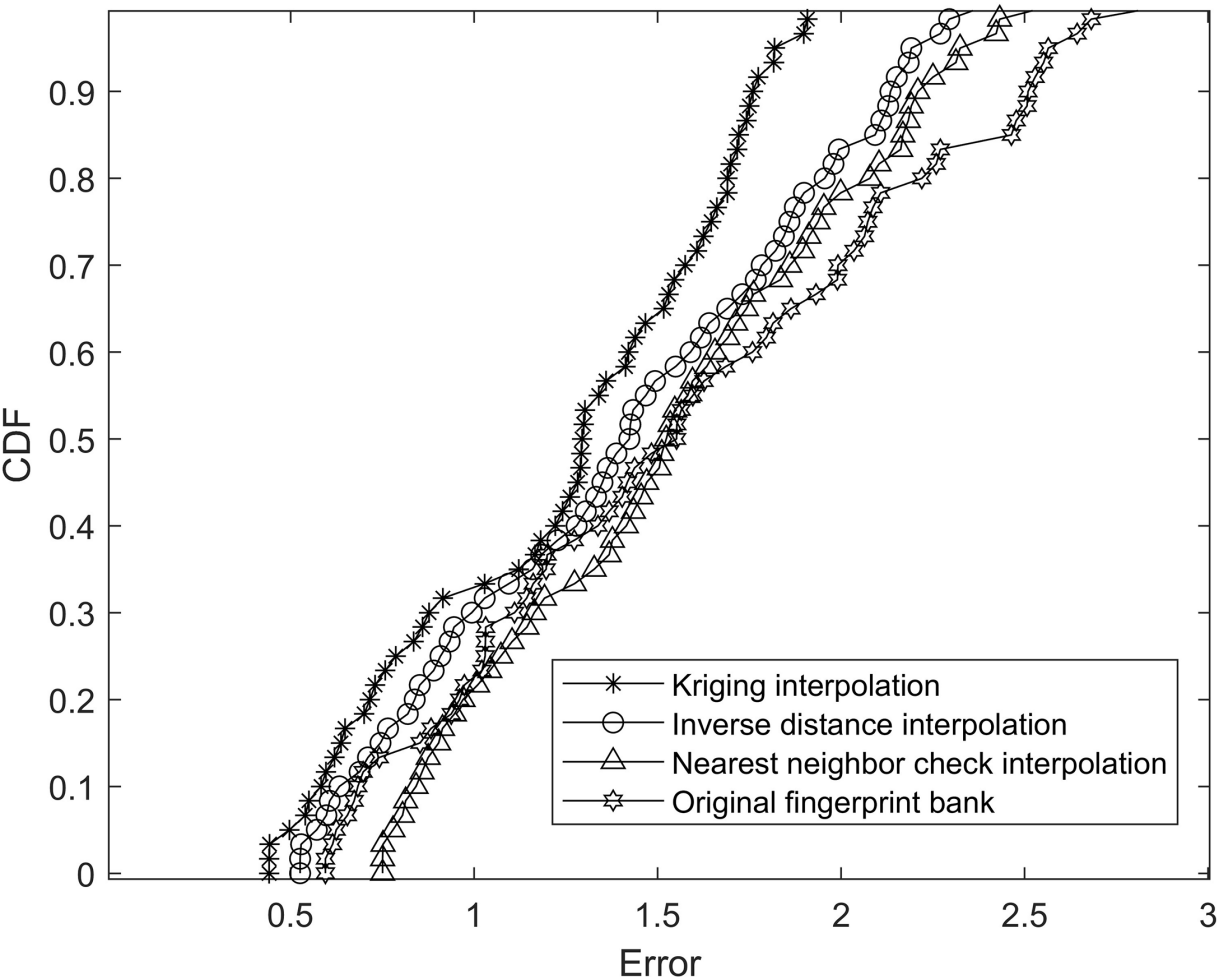
Localization error cumulative distribution function graph of EWKNN under different fingerprint databases.

The EWKNN algorithm is positioned in the fingerprint database and enhanced by the above three interpolation algorithms, and the error probability of the result is statistically analyzed. The cumulative probability error is shown in Fig 9.

Taking the EWKNN algorithm as an example, the fingerprint database enhanced by different interpolation algorithms is located respectively. The results are shown in Fig 9. The positioning error of the Kriging interpolation algorithm is significantly reduced after enhancement, and the effect of the nearest neighbor interpolation algorithm is not apparent. The minimum error positioning result is 0.72m, which is greater than the original fingerprint error. Therefore, this paper chooses the Kriging interpolation algorithm to enhance the fingerprint database.

The Kriging interpolation is used, and the interval size of the fingerprint database is 0.3 m after one interpolation. The same test points are used to test the above fingerprint database and database. Table 4 shows the performance of the above algorithms after interpolating the fingerprint database, and the K value is consistent with the above.

**Table 4:** Positioning error after fingerprint interpolation.

localization algorithm	Average positioning error/ m	Maximum error/ m	Minimum error/ m
KNN algorithm	1.89	3.26	1.06
WKNN algorithm	1.79	2.54	0.61
EWKNN algorithm	1.26	1.95	0.44

It can be seen from Table 4 that after one interpolation, the positioning errors of the above three algorithms are improved. The average positioning error of the KNN algorithm is reduced by 16.5%, the average positioning error of the WKNN algorithm is reduced by 12.6%, and the average positioning error of the EWKNN algorithm is reduced by 21.4%. After one interpolation, the positioning results are improved due to increased fingerprint database density.

**Fig 10:**
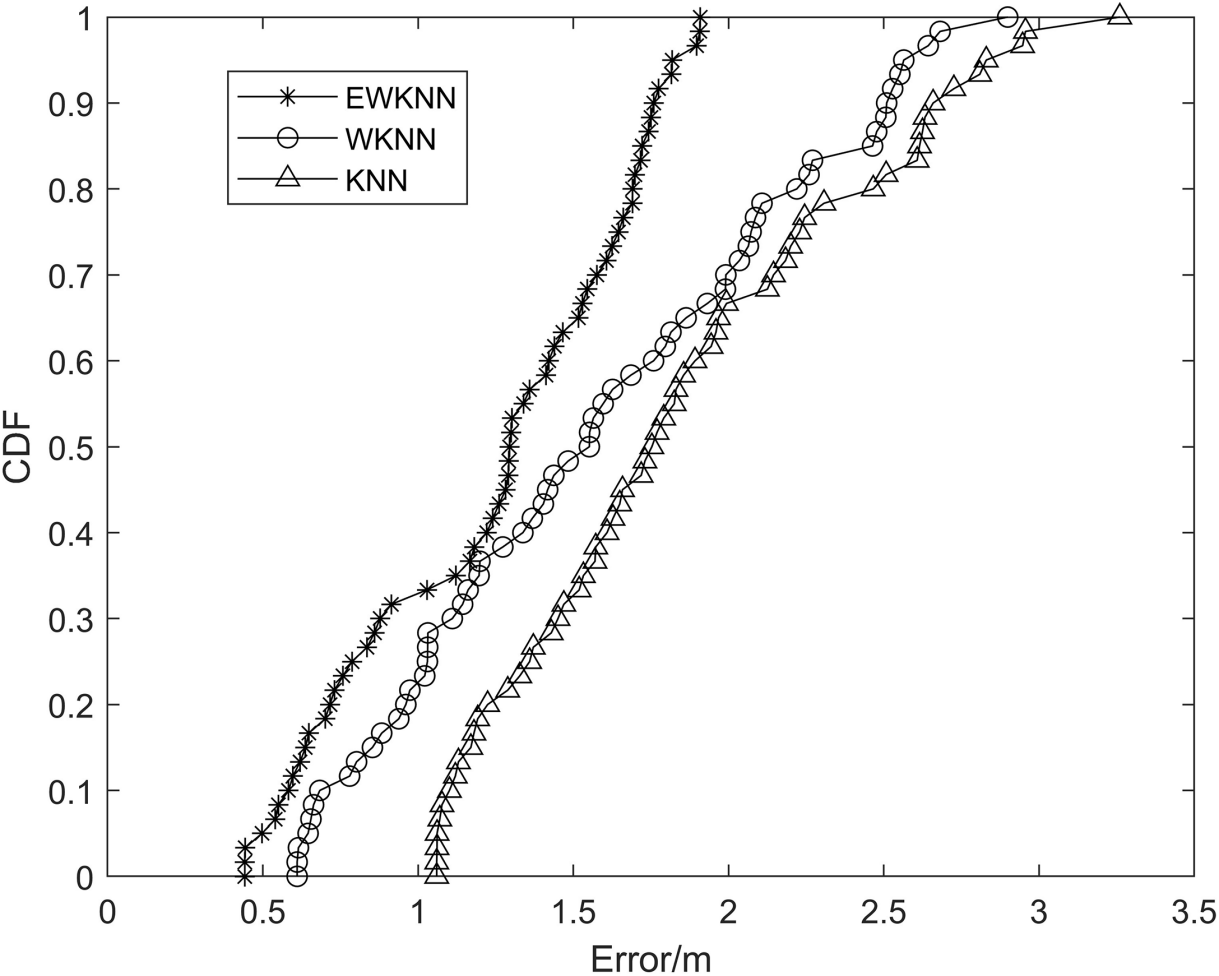
Cumulative distribution function of different algorithms after interpolation.

After interpolation, the error probability statistical analysis of the above three algorithms is carried out, and the cumulative distribution of positioning error is shown in Fig 10. According to Fig 10, the probability of EWKNN algorithm positioning accuracy better than 1.7m is 96%, and the probability of WKNN and KNN algorithm accuracy better than 2m is 89% and 73%, respectively.

### 4.2 Step frequency detection and step size estimation

In the experiment, the tester horizontal hand-held mobile phone parallel to the ground; the experiment was carried out a total of 5 times, every 100 steps, calculate the average error, accuracy and test steps, walking estimation error by the formula (11) to calculate:


$$Error=\frac{\left|test_{step}-ture_{step}\right|}{ture_{step}}\times 100\%$$ (14)

Among them, *Error* is the detection error rate, *test*_*step*_ is the number of steps detected by the detection algorithm, and *ture*_*step*_ is the actual number of steps.

The step results obtained by the peak detection, zero-crossing detection, and the detection algorithm in this paper are tested. The step results of the three algorithms are as follows: Table 5 shows:

**Table 5:** Step frequency detection results The detection results of different step frequency detection algorithms.

Record times	Actual steps	Peak-valley detection		zero-cross detection		proposed method	
		Detection result	error	Detection result	error	Detection result	error
First time	100	106	6	104	4	100	0
Second	100	115	15	97	3	101	1
Third time	100	108	8	105	5	98	2
Fourth time	100	107	7	102	2	101	1
Fifth time	100	109	9	102	2	100	0

It can be seen from Table 5 that the average step frequency detection accuracy of the peak and trough after five-step counting experiments is 91%. The zero-crossing detection algorithm’s average step frequency detection accuracy is 96.8%. The average step frequency detection accuracy of this method is 99.2%. The peak-valley step frequency detection algorithm has the lowest accuracy, mainly because the acceleration peak has pseudo-peaks and pseudo-valleys that the filter does not filter due to noise. The accuracy of the zero-crossing detection algorithm is high, and there is a specific error. This is because there are other zero-crossing moments when the acceleration signal fluctuates wildly when the pedestrian begins to walk. The detection method in this paper has higher accuracy and meets the requirements of indoor positioning for step frequency detection.

The Weinberg [22] model, Kim [23] model, improved Weinberg model [18] and the model proposed in this paper are compared in the step size estimation experiment. In the experiment, a pedestrian walked 50 steps at a fixed length of 60 cm with a mobile phone in front of his chest parallel to the ground. By comparing the experimental results, the model proposed in this paper shows higher stability and minor errors in step size estimation. Specifically, compared with the Kim step model, the average error of the proposed model is reduced by 4.52%. Compared with the Weinberg step size model, the average error is reduced by 3.07%. Compared to the improved Weinberg model, the average error is reduced by 1.13%.These data show that the model in this paper has certain advantages over other models in step size estimation in pedestrian dead reckoning.

**Fig 11:**
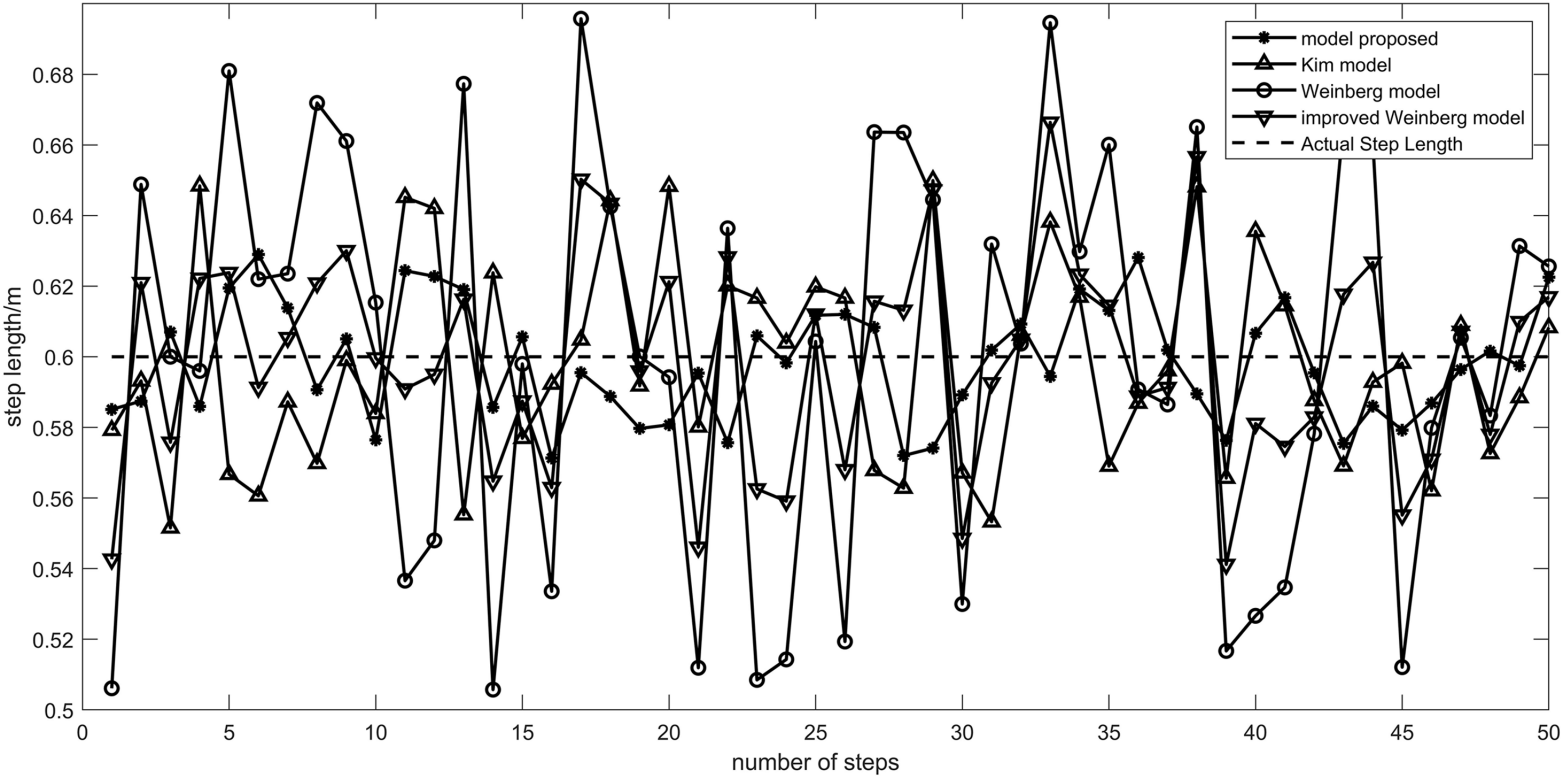
Comparison of the results of one experimental step length. The step length results of four models in one experiment.

**Fig 12:**
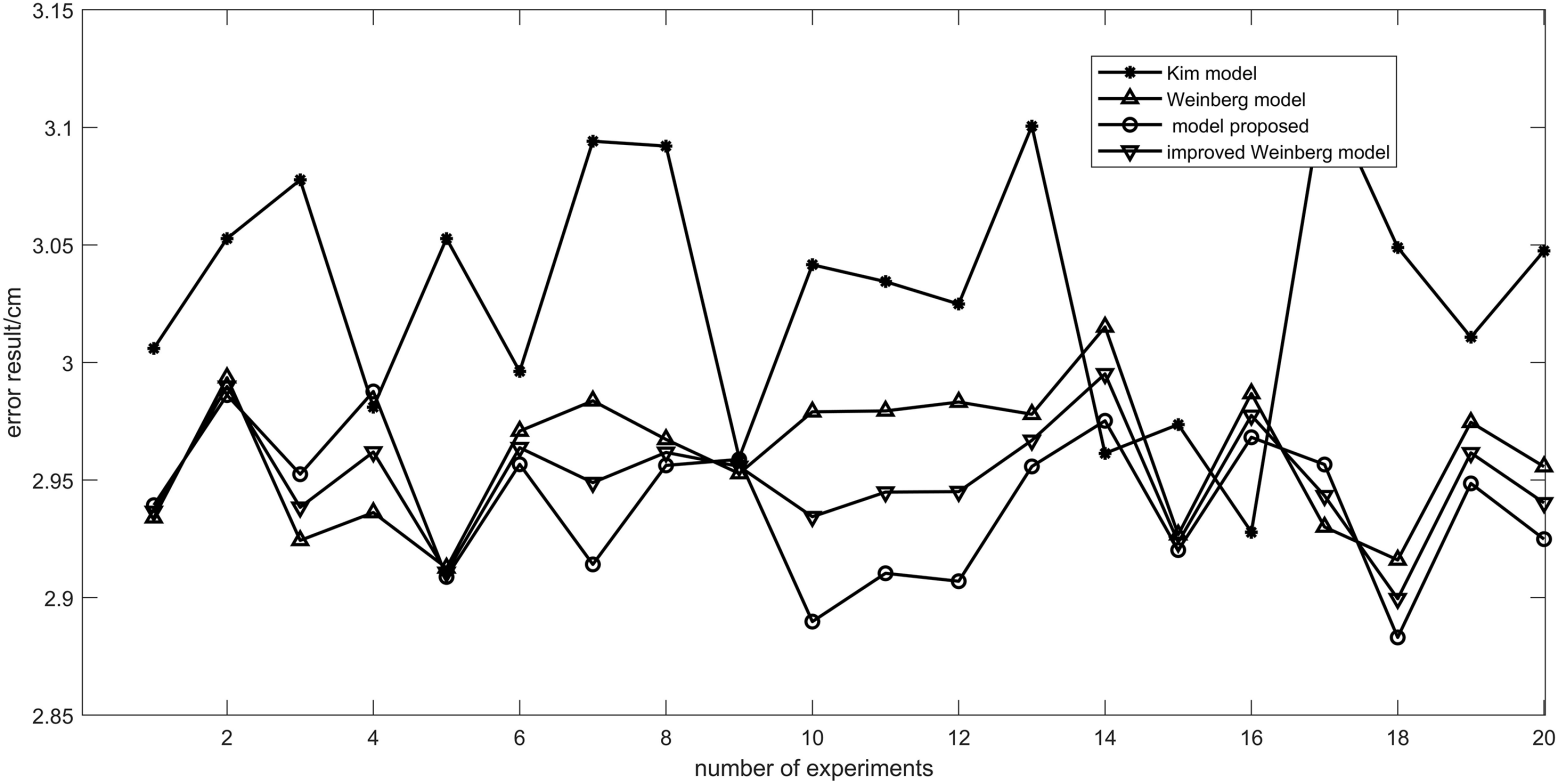
Step error statistical chart. Demonstration of step length errors of four models obtained through 20 experiments.

Fig 11 shows the step size of each algorithm by comparing the step size of each step. Fig 12 shows the average error results of each step experiment obtained by 20 experiments of different models. The algorithm in this paper is more stable than the other two algorithms. The average error of the algorithm in this paper is 2.84 cm 20 times, 3.03 cm for the Kim model, 2.95 cm for the improved Weinberg model, and 2.96 cm for the Weinberg model.

### 4.3 Heading estimation experiment

**Fig 13:**
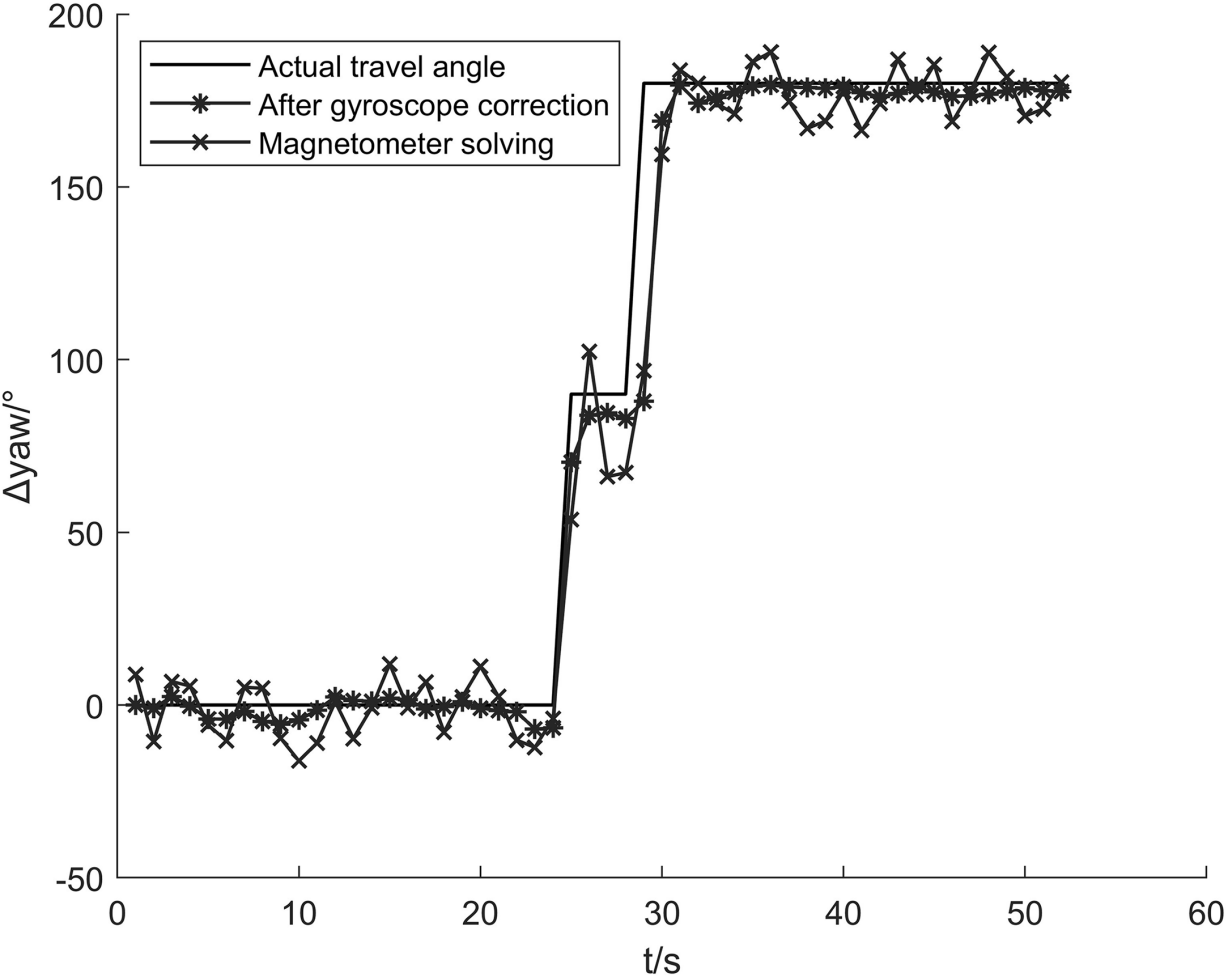
Comparison of heading-angle calculation results. Comparison of heading-angle changes before and after correction.

In this paper, the gyroscope’s high precision n in a short period is used to constrain the heading angle obtained by the magnetometer and the acceleration. Since the gyroscope’s direct calculation is that the carrier coordinate system needs to be transformed through the coordinate system to obtain the angle in the navigation coordinate system, this paper proposes to use the variation of the gyroscope in a short period to correct the heading angle calculated by the magnetic force. The gyroscope detection time window is 1 second, and the heading angle calculation result is shown in Fig 13.

**Fig 14:**
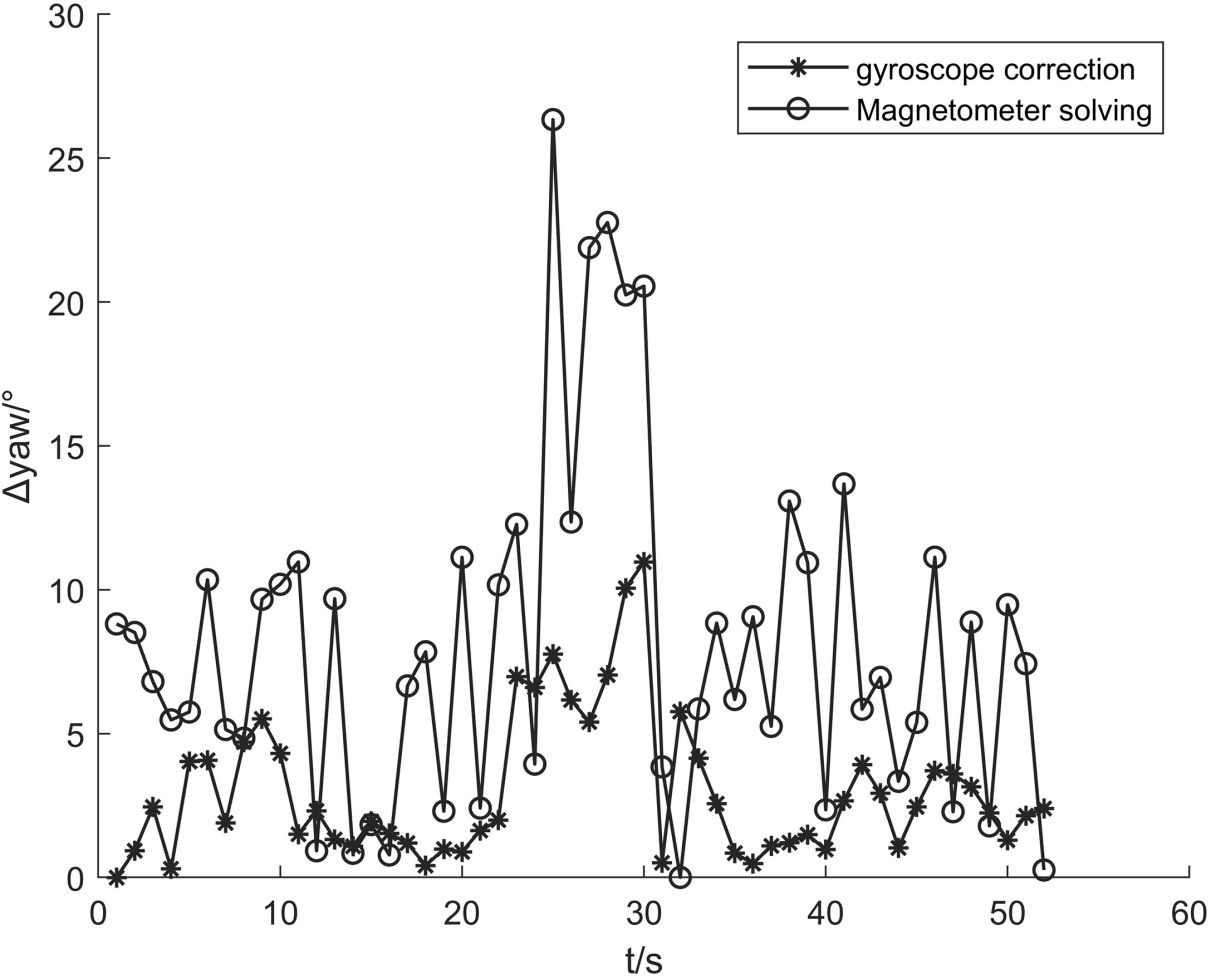
Heading-angle error statistics. Comparison of heading-angle errors before and after correction.

Fig 14 shows that the magnetometer may be distorted when the magnetic force is disturbed when calculating the heading angle, which will cause a significant error in the calculation results and affect the positioning results. The heading angle corrected by the gyroscope is relatively stable, providing stable heading data for PDR.

In order to quantitatively compare the advantages and disadvantages between the algorithm in this paper and the heading angle calculated only by magnetometer, this paper uses Formula (12) to compare the two. The principle of comparison is to extract the absolute value of the difference between the two algorithms and the actual heading angle at different times and compare the maximum error, minimum error, mean value, and standard deviation of the absolute value of the difference to describe the advantages and disadvantages of the two quantitatively.


$$\Delta yaw_{i}=\left|yaw_{T}^{i}-yaw_{D}^{i}\right|$$ (15)

**Fig 15:**
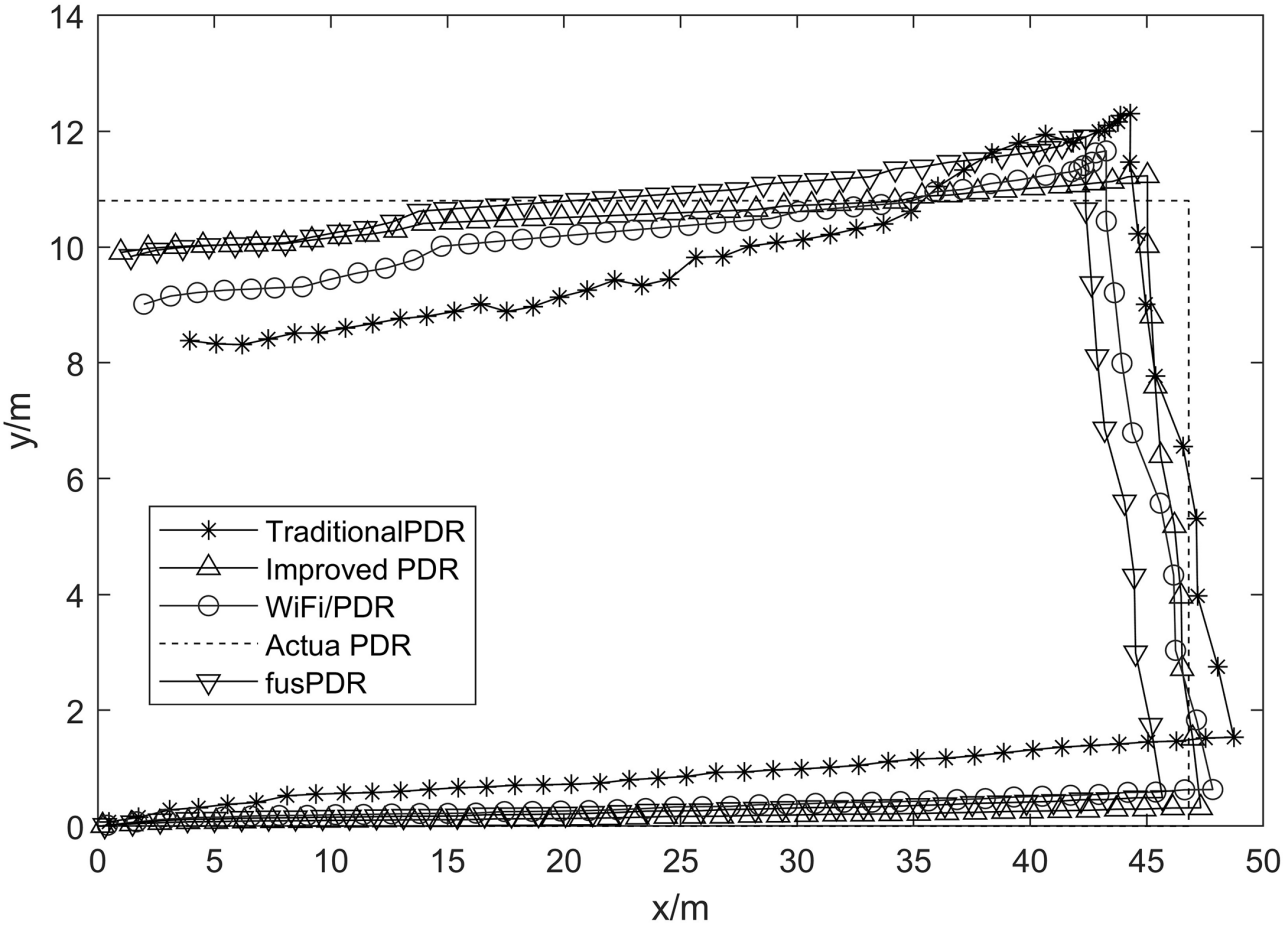
Fusion-based positioning trajectory map. It shows the changes in pedestrian movement trajectories of different algorithms.

Among them, *Δ yaw*_*i*_ is the heading angle difference of each point, $yaw_{T}^{i}$ is the actual heading angle of each point, and $yaw_{D}^{i}$ is the detection heading angle of each point. Fig 15 shows the heading angle error of each point. It can be seen that the algorithm in this paper has a certain inhibitory effect on the magnetometer error. The x-axis is the walking time, and the y-axis is the absolute value of the heading angle error.

The heading angle error fluctuation after the gyroscope constraint is more concentrated. Table 6 compares the performance of the two solution methods from the four aspects of maximum error, minimum error, mean value, and standard deviation. The gyroscope has a good effect on suppressing the heading angle error caused by the abnormal fluctuation of the magnetometer. The mean error is reduced by 40.79%, and the standard deviation of the error is reduced by 45.40%.

**Table 6:** Performance comparison of different solution methods Comparison of Heading Angle Errors Before and After Constraints.

Solution method	Minimum error	Maximum error	Mean value	standard deviation
Magnetometer solution	0.02	26.34	8.14	5.88
proposed method	0	10.97	4.82	3.21

### 4.4 Fusion positioning experiment

The obtained step length, step frequency, and heading angle data can be applied to the PDR algorithm for pedestrian position estimation. In order to reduce the error of PDR, the PDR algorithm in this paper is fused with Wi-Fi fingerprint positioning. The heading angle is calculated using the heading angle calculation method introduced in Section 4.3, the magnetometer combined with the gyroscope azimuth angle calculation, and the average error and cumulative distribution function are used to evaluate the positioning performance.

The tester walked along the established reference route, recorded the inertial data, calculated the PDR positioning results, and recorded the Wi-Fi signal. The Wi-Fi signal was used to provide the initial position and drift suppression for the PDR and repeated several times to reduce the random error. Fig 15 compares the positioning trajectory results of the conventional PDR, the improved PDR, fusPDR, and the proposed WiFi/PDR algorithm.

The PDR algorithm has inherent systematic errors in estimating step length and frequency. These errors will accumulate with the increase in walking distance, resulting in an increasing deviation between the calculated and actual walking trajectories. However, this paper combines the Wi-Fi fingerprint positioning results into the PDR by combining the extended Kalman filter (EKF), which significantly reduces the error accumulation and makes the estimated trajectory closer to the natural walking trajectory, thus improving the accuracy of trajectory estimation. This method that combines Wi-Fi positioning results effectively reduces error accumulation and improves the accuracy of trajectory estimation.

**Fig 16:**
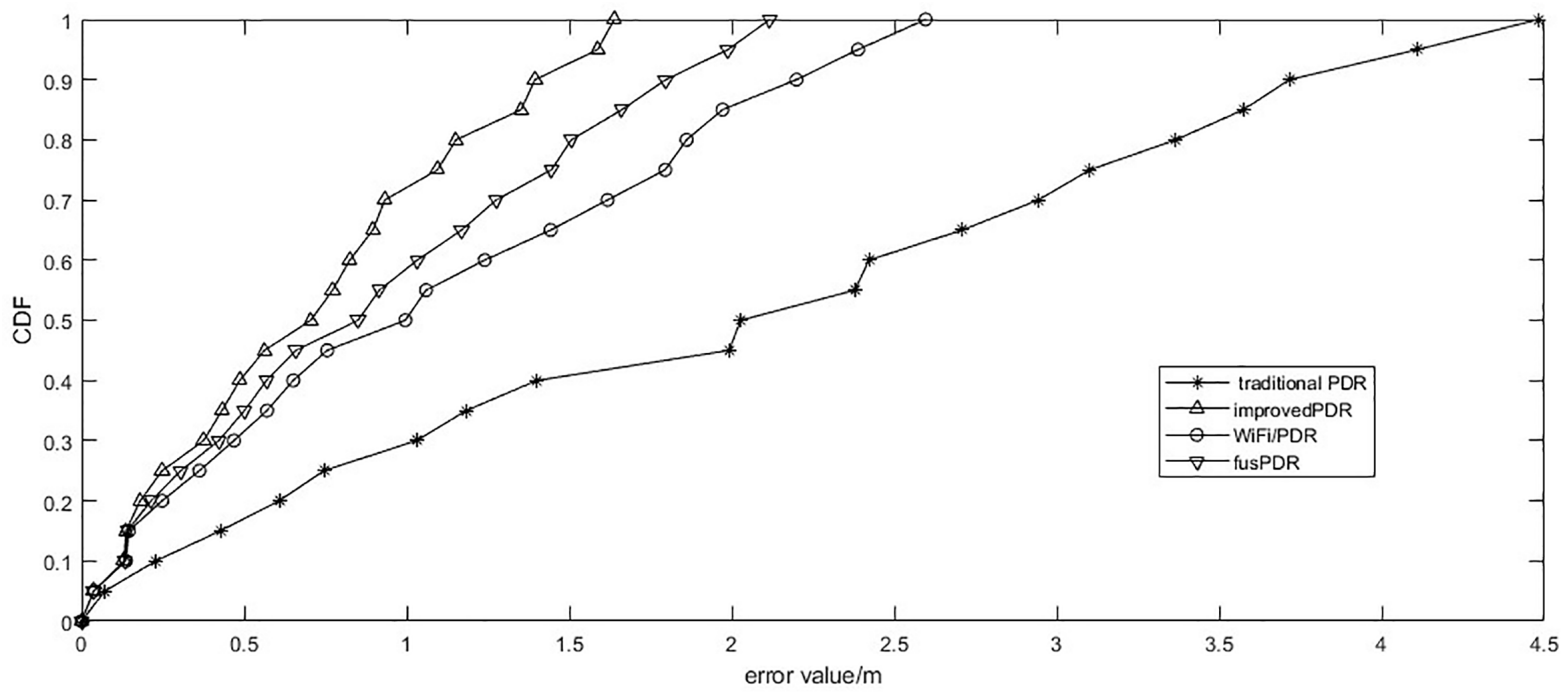
Error comparison of different positioning methods. Cumulative distribution function (CDF) comparing four algorithms.

Fig 16 is the cumulative distribution curve of positioning error of the Wi-Fi fusion PDR algorithm, improved PDR, fusPDR and traditional PDR algorithm in the positioning process.

According to the statistical data, the average positioning error of the traditional PDR algorithm is 2.02 meters, and 90% of the positioning error is less than 3.71 meters. The average positioning error of the improved PDR algorithm is 1.07 meters, and 90% of the positioning error is less than 2.12 meters. The fusPDR [8] algorithm achieves an average positioning error of 0.89 m, with 90% of positioning errors being less than 1.8 m. The average positioning error of the Wi-Fi and improved PDR fusion indoor positioning algorithm is reduced to 0.71 meters, and 90% of the positioning error is less than 1.42 meters. This shows that the Wi-Fi-corrected PDR algorithm significantly improves positioning accuracy and stability.

## 5. Conclusion

This paper proposes a fusion algorithm that integrates Wi-Fi fingerprint positioning with an improved Pedestrian Dead Reckoning (PDR) method to enhance indoor positioning accuracy. The algorithm incorporates a dual-gyroscope constrained magnetometer approach, which improves the robustness and stability of heading angle estimation even under magnetic disturbances. In addition, an improved step frequency detection method based on acceleration zero-crossing and peak-valley pairing, together with Kriging interpolation of the fingerprint database, enables high-precision step counting and enhances the performance of KNN, WKNN, and EWKNN algorithms. Compared with traditional PDR and existing models such as Weinberg and Kim, the proposed method significantly reduces the average positioning error. The final experimental results demonstrate that the fusion algorithm achieves noticeable improvements in both accuracy and stability, validating its effectiveness and highlighting its potential for high-precision smartphone-based indoor positioning applications.

## Supporting information

S1 File Supplementary experimental data and MATLAB simulation results supporting the Wi-Fi/PDR fusion algorithm. (ZIP)
